# Preference by the Nymphs of *Americabaetis alphus* Lugo-Ortiz & McCafferty, 1996 (Baetidae: Ephemeroptera) for Feeding substrate and Food Size Under Laboratory Conditions

**DOI:** 10.1007/s13744-026-01361-2

**Published:** 2026-02-20

**Authors:** Bárbara Oleinski, Thais Carneiro, Laís Olivera das Neves, Mikael Luiz Pereira Morales, Edélti Faria Albertoni

**Affiliations:** 1 Programa de Pós-graduação em Biologia de Ambientes Aquáticos Continentais, Instituto de Ciências Biológicas (ICB), Universidade Federal do Rio Grande (FURG), Rio Grande, Rio Grande do Sul Brazil; 2 Programa de Pós-graduação em Oceanologia, Instituto de Oceanografia (IO), Universidade Federal do Rio Grande (FURG), Rio Grande, Rio Grande do Sul Brazil; 3 Programa Institucional de Pós-Doutorado da Coordenação de Aperfeiçoamento de Pessoal de Nível Superior (PIPD-CAPES/PPGO), Rio Grande, Brazil

**Keywords:** Feeding habits, Aquatic invertebrates, Immatures, Periphyton, Particulate organic matter

## Abstract

Feeding by mayflies is influenced by resource availability, and their feeding preferences can vary acrossdevelopment stages. Studies assessing food preferences can therefore provide insights into the functional role of a species. The aim of this study was to evaluate the feeding preferences of *Americabaetis alphus* nymphs on periphyton growing two different substrates and two sizes of particulate organic matter. Experimental units were composed of acrylic and stainless steel substrates; the control contained no nymphs. The substrate preference was determined by the presence or absence of nymphs on the substrates. Bacterial density and chlorophyll-a concentration were measured to evaluate food intake, and the frequency of microbial taxa adhering to the substrates was calculated. *Salix humboldtiana* leaves were processed into two categories, fine particulate organic matter (FPOM) and coarse particulate organic matter (CPOM). In each experimental unit, 25 mg of FPOM and 25 mg of CPOM were provided. Food intake was estimated by the difference between the initial and final weights of FPOM and CPOM. The highest bacterial and chlorophyll-*a* intake was observed on the stainless steel substrates. The taxonomic composition differed between the substrates, with stainless steel showing a higher frequency of diatoms. In the second experiment, the weights of FPOM and CPOM were reduced after the feeding period, with a greater reduction observed in FPOM. It is important to evaluate resource preferences in a combined manner, as well as to assess other Baetidae species, given the scarcity of information on the topic for the family.

## Introduction

The mayflies (Ephemeroptera) are widely distributed geographically (Da-Silva and Salles 2024), colonizing diverse freshwater environments (lentic or lotic) and brackish waters (Sartori and Brittain 2015). The variability of morphological, physiological, and behavioral functional traits among species within this order allows a wide distribution of organisms across these systems (Jacobus et al. 2019). Ephemeropteran nymphs are mainly herbivorous or detritivores (Sartori and Brittain 2015), with their main food resources being fine particulate organic matter (FPOM), coarse particulate organic matter (CPOM), and periphytic, epilithic, and filamentous algae (Domínguez et al. 2023), however, filtering and predatory species also occur (Merritt & Cummins 1996). Nymphs play a role in nutrient cycling by processing organic matter, and they are considered good biological indicators, as some species are sensitive and intolerant to environmental changes (Da-Silva and Salles 2024). They also serve as prey for other organisms (e.g*.*, fish, amphibians, and insects) (Domínguez et al. 2006).

Among the families of Ephemeroptera, Baetidae is one of the most representative in terms of number of species (Edmunds et al. 1976). The family is cosmopolitan, with the exception ofAntarctica and New Zealand (Kaltenbach and Gattolliat 2019). The genus *Americabaetis* is present in several South American countries (including Brazil, Argentina, Chile, Colombia, Paraguay) (Salles et al. 2003; Gutiérrez and Llano 2015; Salinas-Jimenez et al. 2025). In Brazil, species in the genus inhabit intermittent and permanent wetlands in Rio Grande do Sul (Maltchik et al. 2009), as well as lotic environments (Salles et al. 2010) in different regions of the country. *Americabaetis alphus* Lugo-Ortiz & McCafferty, 1996 is one of the most abundant species of Ephemeroptera in Brazil. It is associated with substrates such as leaf litter and marginal vegetation (Salles et al. 2010) and colonizes backwater zones (Salles et al. 2003) as well as rapids.

The diet of nymphal mayflies can be influenced by the availability of food resources, and preference for specific food types can vary depending on the developmental stage of the organism (Shimano et al. 2012). One of the food resources consumed by nymphs is periphyton formed through a successional process that occurs on artificial (e.g*.*, acrylic and stainless steel) and natural substrates (e.g*.*, leaves, rocks, and shells) submerged in aquatic environments (Agostini et al*.*^2018^). The process begins with the adhesion of autotrophic and heterotrophic bacteria that secrete an extracellular polymeric matrix and form a biofilm (Martín-Rodríguez et al. 2015; Agostini et al*.*^2018^). Subsequently, other microorganisms, such as protozoa, fungi, green algae, diatoms, adhere to the biofilm, forming the periphyton (França et al. 2011; Agostini et al*.*^2018^).

Zinc-based substrates, such as stainless steel, may be more attractive for bacterial biofilm formation, since zinc is an essential element in bacterial metabolism (Bong et al. 2010; Agostini et al. 2019). However, zinc at high concentrations can cause toxic effects (Eisler 1998). On plastic substrates, the biological community of the periphyton is more diverse and distinct than that of the surrounding environment (e.g., water and sediment) (Vannini et al. 2021). Thus, plastic surfaces can be more attractive in meeting the nymphal demand for food resources.

In the process of decomposition of particulate organic matter (POM), coarse particulate organic matter (CPOM) is converted into fine particulate organic matter (FPOM) (Gonçalves et al. 2014), a process that occurs through leaching, microbial conditioning, and colonisation by invertebrates (Graça et al. 2005). In aquatic environments, the availability of particle sizes (FPOM and CPOM) varies over time and is differentially ingested by organisms with specifically adapted mouthparts (Shepard and Minshall 1984). Studies have shown that other genera of Ephemeroptera (scrapers and collector-scavengers) exhibit dietary preferences for particle sizes and algal types (Hamilton and Hugh 1983; McShaffrey and McCafferty 1991; Chuang et al. 2014; Banegas et al. 2020). In general, the type of material consumed is related to its availability in the microhabitat where nymphs are feeding (Hamilton and Hugh 1983).

Studies involving food preferences *Americabaetis* and *A. alphus* are scarce in literature. Some studies have shown that scrapers in a community associated with decomposing debris in freshwater environments can accelerate the decomposition process, contributing to the degradation of organic matter (Tonin et al. 2018; Marks 2019; Bao et al. 2023). Assessing feeding behaviors will help to understand the role of these organisms in the ecological processes of matter cycling in freshwater ecosystems (Jacobus et al. 2019).

The objective of this study was to evaluate the feeding preferences of *A. alphus* nymphs for periphyton developed on two substrates (stainless steel and acrylic) and for two sizes of particulate organic matter (FPOM and CPOM). This approach was based on the previous evidence indicating that substrate type influences periphyton composition, potentially affecting food availability for grazing invertebrates, and that plastic substrates often support more diverse biological communities than metal substrates. Additionally, particle size is known to influence ingestion by Ephemeroptera nymphs due to differences in mouthpart morphology and feeding strategies. Therefore, we tested the following hypotheses: (1) *A. alphus* nymphs would preferentially feed on periphyton adhering to acrylic rather than stainless steel when offered simultaneously; and (2) *A. alphus* nymphs would preferentially consume FPOM rather than CPOM when both particle sizes are simultaneously available. Understanding the feeding preferences of *A. alphus* nymphs in relation to substrate type and organic matter size provides insights into the trophic role of this species in freshwater ecosystems.

## Material and methods

### Sampling and acclimatization of nymphs

Nymphs of *A. alphus* were collected in May 2024 from an urban temporary puddle at the Carreiros Campus of the Federal University of Rio Grande (FURG), located in the municipality of Rio Grande, Rio Grande do Sul, Brazil (32°04′20.3" S 52°09′38.2" W). The organisms were identified using literature specific to South American Ephemeroptera (Domínguez et al. 2006), and specimens were deposited in the Limnology Laboratory of the Institute of Biological Sciences at the same university. The nymphs were captured with a 68-μm mesh net until the sample size necessary for the experiments (n = 50 per experiment) was reached. In the laboratory, nymphs measuring 3–4 mm in length at an intermediate nymphal stage were selected (adapted from Banegas et al. 2020). The individuals were kept in plastic containers in the incubation laboratory with a photoperiod of 12 h light and 12 h dark and a controlled temperature of 25 ± 0.1ºC. The containers were filled with continuously aerated distilled water, and the nymphs were fed ad libitum with a commercial fish feed (Alcon Basic; Alcon Pet), formulated from dehydrated spirulina, fish meal, and soybean meal. The water in the containers was changed 24 h prior to performing to the experiments, and the organisms were subsequently starved.

### Experimental design

## Experiment 1: Acrylic and stainless steel substrates

Rectangular acrylic and stainless steel substrates (plates) with an area of 12.5 cm^2^ were placed in the water column of an urban lake using ropes and buoys. The substrates were exposed for one month to allow for periphyton development. The substrates were collected 24 h prior to use in the experiment, stored in plastic containers with lake water, and kept in the laboratory under a photoperiod of 12 h light and 12 h dark and a controlled temperature of 25 ± 0.1ºC.

**Fig. 1:**
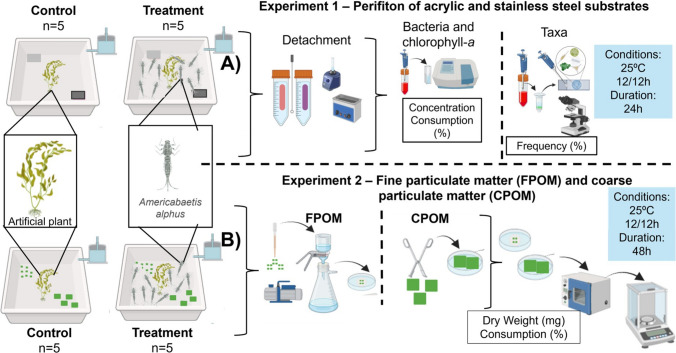
Experimental design scheme. **A**) Experiment 1 – Feeding preference on periphyton adhered to acrylic and stainless-steel substrates. **B**) Experiment 2 – Feeding preference between fine particulate organic matter (FPOM) and coarse particulate organic matter (CPOM)

Five substrates of each type were selected to determine the initial parameters of bacterial biomass, chlorophyll-a concentration, and frequency (%) of adhering taxa. The experiment consisted of a treatment and a control with five replicates each (Fig. 1A). Each experimental unit of the treatment was composed of 2 L of continuously aerated distilled water, a 10-cm tall artificial plant, one acrylic and one stainless-steel substrate, and 10 nymphs of *A. alphus*. The control had the same composition except for the absence of nymphs. The incubation room was kept at a controlled temperature of 25 ± 0.16 ºC, and the experiment lasted for 24 h. The preference for substrate type was assessed by counting the number of nymphs present on the underside of each substrate at 1, 2, 4, and 24 h.

Immediately after counting nymphs at 24 h, the acrylic and stainless steel substrates from the treatment and the control were washed three times with sterile distilled water (autoclaved and filtered) to eliminate possible planktonic organisms (Agostini et al. 2021) and stored individually in 50-mL Falcon tubes containing sterile distilled water. For the detachment of the periphyton and suspended biological material, the substrates were individually scraped with sterile microbiological loops and agitated in a vortex for 1 min. Subsequently, the substrates were placed in an ultrasonic bath (40 kHz) for 2 min and vortexed for 1 min (adapted from Agostini et al. 2021). This procedure was performed for both the treatment and control substrates.

From each replicate, three aliquots of 1 mL each were removed from each Falcon tube and used to determine planktonic bacterial biomass (Fig. 1A) using a spectrophotometer at 630 nm (Morales et al. 2024). The use of the spectrophotometer in this analysis is based on the principle of turbidimetry and the linear relationship between bacterial biomass and optical density. The values for each replicate and substrate type were expressed as absorbance. We considered the final control reading to be 100% and calculated the bacterial consumption according to the formula:

$$\text{BC }\left(\mathrm{\%}\right)=100-((\mathrm{FR}*100)/\mathrm{AFR})$$

where BC = bacterial consumption, FR = final reading, AFR = average final reading.

To analyze the concentrations and consumption of chlorophyll-a, aliquots of 5 mL were removed from each Falcon tube and centrifuged at 4000 rpm for 10 min; the supernatant was discarded (Morales et al. 2024). Then, 4 mL of methanol (99.99%) was added to each tube, and the samples were kept in the dark under refrigeration. The samples were later centrifuged again, and the optical density was measured in a spectrophotometer at 663 and 750 nm (Mackinney 1941). The consumption of chlorophyll-a was determined using the formula described above for BC (%), with the optical density values replacing the absorbance readings.

A 1-mL aliquot from each Falcon tube was transferred to an Eppendorf tube containing 200 μL of 4% formaldehyde to fix the samples and halt the growth of the microorganisms (Morales et al. 2024) and to determine the taxa present. Three temporary slides were made from each sample and viewed under an Olympus CX41 microscope at 100_X_ magnification with immersion oil. On each slide, 10 randomly selected points were used to determine the taxa frequency (%) (adapted from Boyero et al. 2020) of green algae, bacteria, cyanobacteria, and diatoms. Bacteria were identified by visualizing their morphotypes (e.g., cocci, rods, among others) (adapted from Liu et al. 2001; Smeda-Pienaar et al. 2017), and the other taxa were identified based on Lopretto & Tell (1995). Frequency was determined for each plate and treatment.

## Experiment 2: Particulate Organic Matter (POM)

*Salix humboldtiana* Willd. (Salicaceae) was selected to investigate the food preference of *A. alphus* nymphs between fine particulate organic matter (FPOM) and coarse particulate organic matter (CPOM), the plant species was chosen based on its wide distribution in the region and its role in the entry of organic matter into aquatic environments (Telöken et al. 2011). To obtain FPOM and CPOM, the leaves of *S. humboldtiana* were washed, brushed, and stored in an oven at 60 ºC until dry biomass was obtained. Subsequently, the leaves were cut into specific sizes to obtain FPOM ranging from 500 and 1000 μm and CPOM larger than 1000 μm. At 24 h prior to the experiment, the FPOM and CPOM were hydrated separately with distilled water according to the protocol described by Banegas et al. (2020) with modifications. Soaking in water softens the tissues and causes the particles to settle at the bottom, making them available to the nymphs.

The experiment consisted of a treatment and a control with five replicates each (Fig. 1B). Each replicate of the treatment consisted of 2 L of distilled water, a 10-cm tall artificial plant, 25 mg of FPOM and 25 mg of CPOM, and 10 *A. alphus* nymphs. The control replicates had the same composition except for the absence of nymphs. All experimental units were continuously aerated and kept in an incubation laboratory with a photoperiod of 12 h light and 12 h dark at a controlled temperature of 25 ± 0.32 ºC. The exposure time lasted 48 h, after which the nymphs were counted and collected. The FPOM was collected with a Pasteur pipette (Banegas et al. 2020) and filtered through a previously dried and weighed filter according to Paranhos (1996). The CPOM was collected with tweezers and deposited in Petri dishes. The samples were dried in an oven at 60 ºC and weighed. The consumption of FPOM and CPOM was determined by the difference: Fw – Iw, onde: Fw = Final weight; Iw = Initial weight (Banegas et al. 2020).

### Data analysis

To detect significant differences between the treatment and the control or among observation times, the generalised linear model was used with a binomial distribution for the preference data (number of choice frequencies) by substrate type, as this variable is discrete, that is, the count of choices (successes – preference) in relation to the total number of observations (trials). Because bacterial biomass, chlorophyll-a concentration, and weight of FPOM and CPOM are continuous variables, the normality and homoscedasticity of the residues were verified by the Shapiro–Wilk and Levene tests, respectively. With the acceptance of the premises, a two-way ANOVAwas used. When the significant effect of a variable was detected, a post-hoc Tukey comparison was performed to detect significant differences between the levels of the factors. The t-test was used to detect any significant difference in consumption of FPOM and CPOM between the treatment and the control, as well as for bacterial and chlorophyll consumption. The statistical analyses were performed using the GraphPad Prism 8.4 software.

## Results

### Experiment 1: Acrylic and stainless steel substrates

**Fig. 2:**
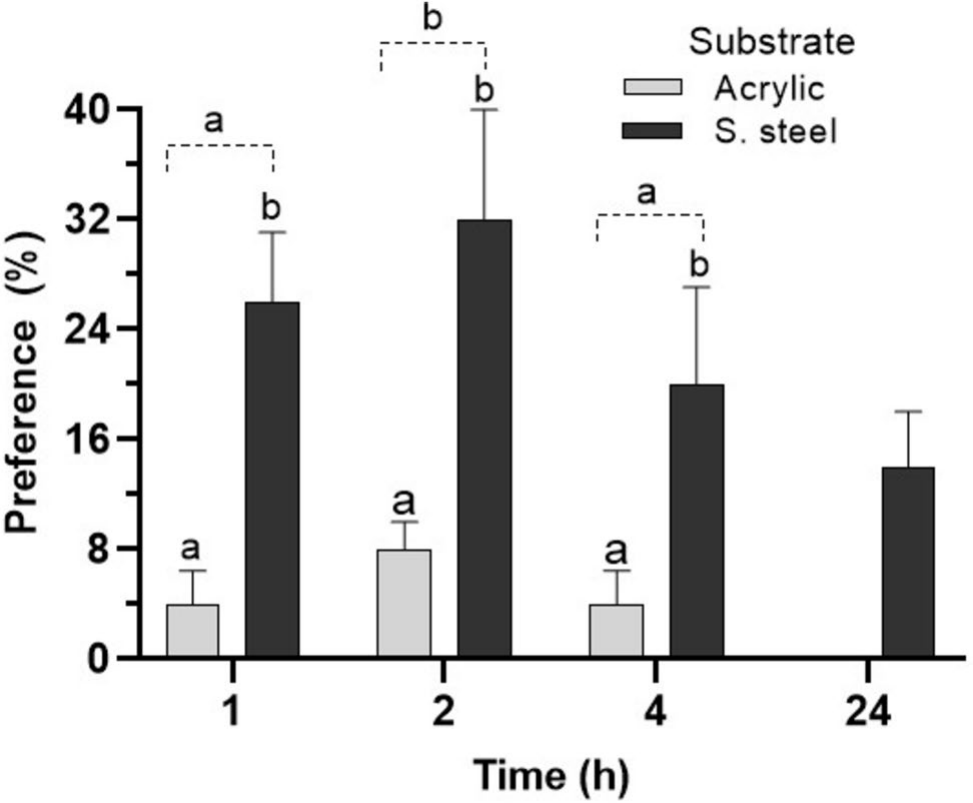
Mean ± SD preference (%) of nymphal *Americabaetis alphus* on acrylic and stainless steel substrates at different times (h) of observation. Means with different letters within each observation time are significantly different between substrates (*p* < 0.05). Means with different letters within each time interval (dotted line) are significantly different among observation times (*p *< 0.05)

The nymphs of *A. alphus* showed a feeding preference for periphyton on the stainless steel substrate (SSS) at 1 (*p *= 0.008, q = 3.05), 2 (*p* = 0.003, *q* = 3.66), and 4 h (*p* = 0.008, *q* = 2.94) of observation (Fig. 2). At 24 h, no nymphs were observed under the acrylic substrate (AS), therefore, it was not possible to statistically compare this time with the other observation times or with the SSS (Fig. 2). When comparing only the observation times (without considering the different substrates), the preference was equal at 1 (*p* = 0.990, q = 0.69) and 4 (*p *= 0.990, *q *= 0.75), with a significant difference only at 2 h (1 vs 2—*p* = 0.044, q = 2.54; 2 vs 4—*p* = 0.048, *q *= 2.81) (Fig. 2).

**Fig. 3:**
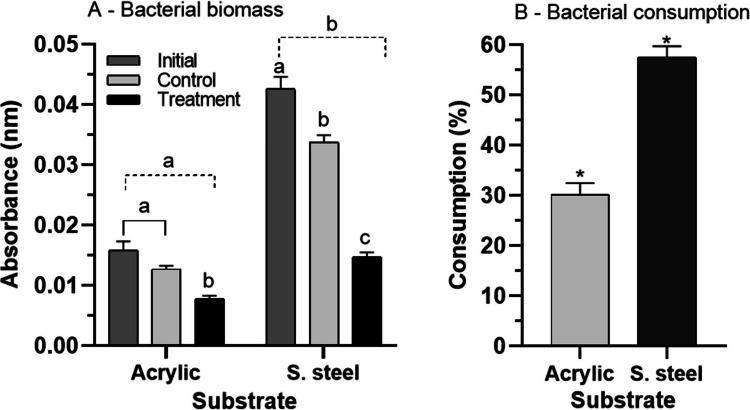
Mean ± SD of bacterial biomass (**A**) and bacterial consumption (**B**) on acrylic and stainless steel substrates exposed to nymphs of *Americabaetis alphus*. In "A": Different letters within each substrate – shows statistical difference between treatment and control, and time (*p *< 0.05); Different letters for each substrate (dotted stroke) – shows statistical difference between substrates (*p* < 0.05). In B, * = significant difference between substrates (*p* < 0.05)

The SSS had significantly higher (*p *= 0.0001,* q* = 15.96) bacterial biomass than the AS at the initial time (T_0_) in the control (no exposure to individuals) and the treatment (with exposure to individuals) (Fig. 3A). SSS showed significantly lower bacterial biomass compared to the initial biomass (*p* = 0.0001, *q* = 25.99) and the control biomass (*p *= 0.0001, *q *= 20.42) (Fig. 3A). The same pattern was found for the biomass of the AS with the control (*p* = 0.001, *q* = 5.22) and the initial biomass (*p* = 0.0001, *q* = 7.46) (Fig. 3A). Bacterial consumption was higher on the SSS (57.5 ± 7.9%) than on the AS (30.2 ± 8.4%) (*p* = 0.0001, *t* = 8.81) (Fig. 3B).

**Fig. 4:**
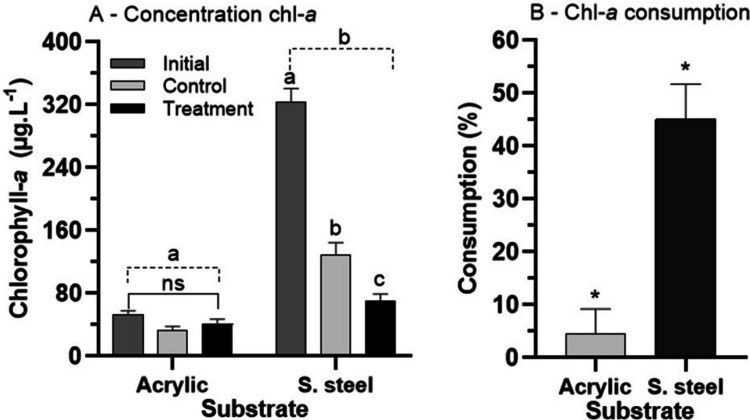
Mean ± SD of chlorophyll-a concentration (**A**) and chlorophyll-a consumption (**B**) on acrylic and stainless steel substrates exposed to nymphs of *Americabaetis alphus*. In A, means with different letters within each substrate are significantly different (*p *< 0.05) between treatment and control and among observation times; Different letters for each substrate (dotted line) – shows statistical difference between substrates (*p* < 0.05); NS = no significant difference (*p* > 0.05). In B, * = significant difference between substrates (*p *< 0.05)

The initial concentration of chlorophyll-a was significantly higher (*p *= 0.0001, q = 17.95) on the SSS than on the AS (Fig. 4A). The concentrations of chlorophyll-a on the AS were similar at T_0_ (*p* = 0.4920, q = 1.64) and at the end of 24 h (*p* = 0.7687, q = 0.99) in the control and the treatment (*p* = 0.8887, *q* = 0.66) (Fig. 4A). There was a significant decrease in the concentration of chlorophyll-a on the SSS in the control (*p* = 0.0001,* q *= 15.62) and treatment (*p* = 0.0001, *q *= 20.27) after 24 h compared to T_0_ (*p* = 0.0122, *q* = 4.64) (Fig. 4A). The consumption of chlorophyll-a was higher on the stainless steel (45.1 ± 8.3%) than on the acrylic (4.6 ± 4,2%) (*p *= 0.0011, *t* = 5.00) (Fig. 4B).

**Fig. 5:**
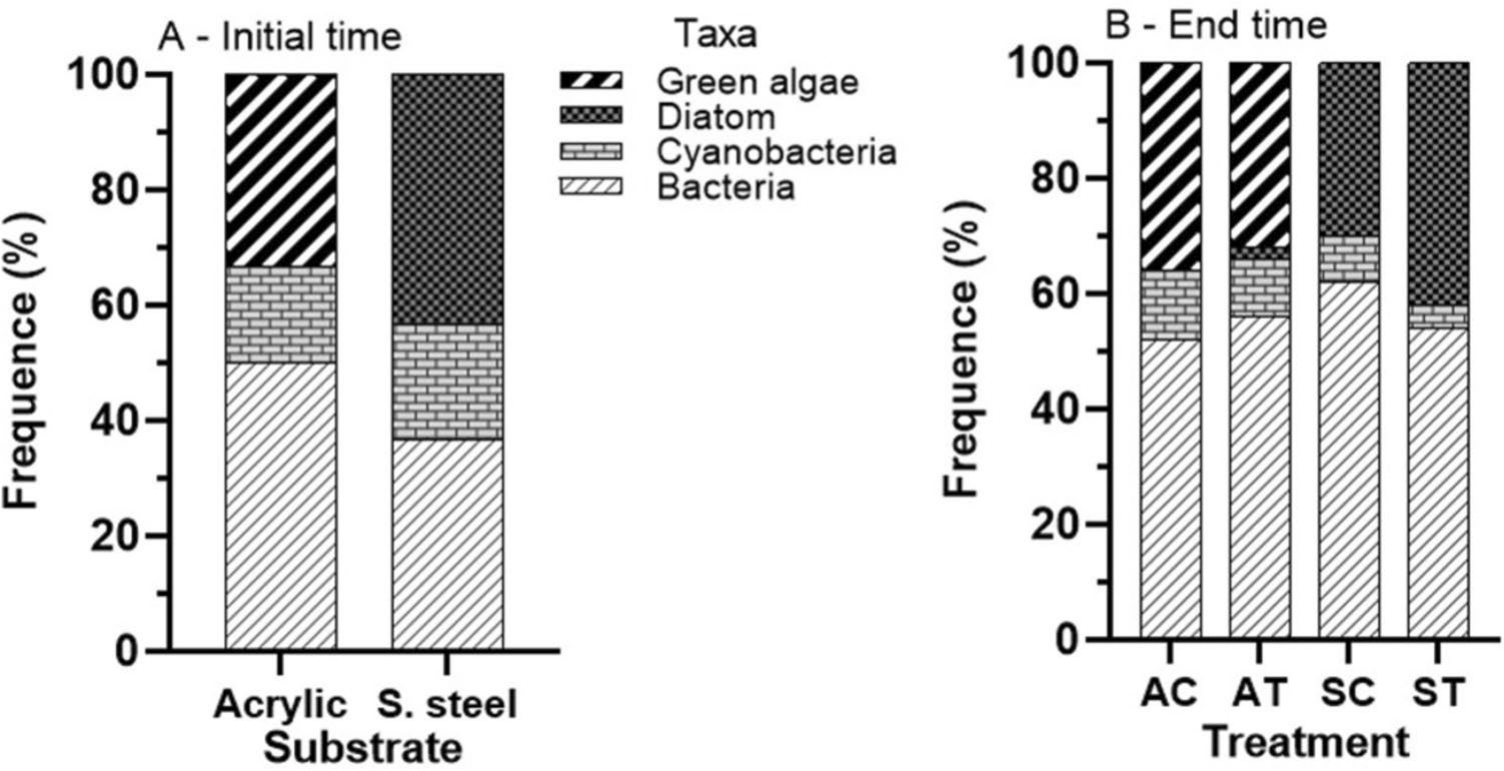
Frequency (%) of taxa in the periphyton on acrylic and stainless-steel substrates exposed to nymphs of *Americabaetis alphus*. **A**) At initial time of exposure; **B**) At 24 h after with exposure. AC = unexposed acrylic in the control; AT = exposed acrylic in the treatment; IC = unexposed stainless steel in the control; IT = exposed stainless steel in the treatment

At T_0_ on the acrylic, bacteria were the most frequent (50.0%), while cyanobacteria were the least frequent (12.0%) (Fig. 5A). On the stainless steel at T_0_, diatoms were more frequent (43.3%) and cyanobacteria less (20.0%) (Fig. 5A). On the acrylic at 24 h, a high frequency of bacteria in the control and the treatment (52.0 and 56.0%, respectively) and a low frequency of cyanobacteria in the control (12.0%) and diatoms in the treatment (2.0%) were observed (Fig. 5B). The exclusive occurrence of green algae on the acrylic was observed at T_0_ and at 24 h in the control (Fig. 5A, B). Diatoms were exclusively present on the stainless steel at T_0_ and 24 h in the control and the treatment (Fig. 5A, B).

## Experiment 2: Particulate Organic Matter (POM)

**Fig. 6:**
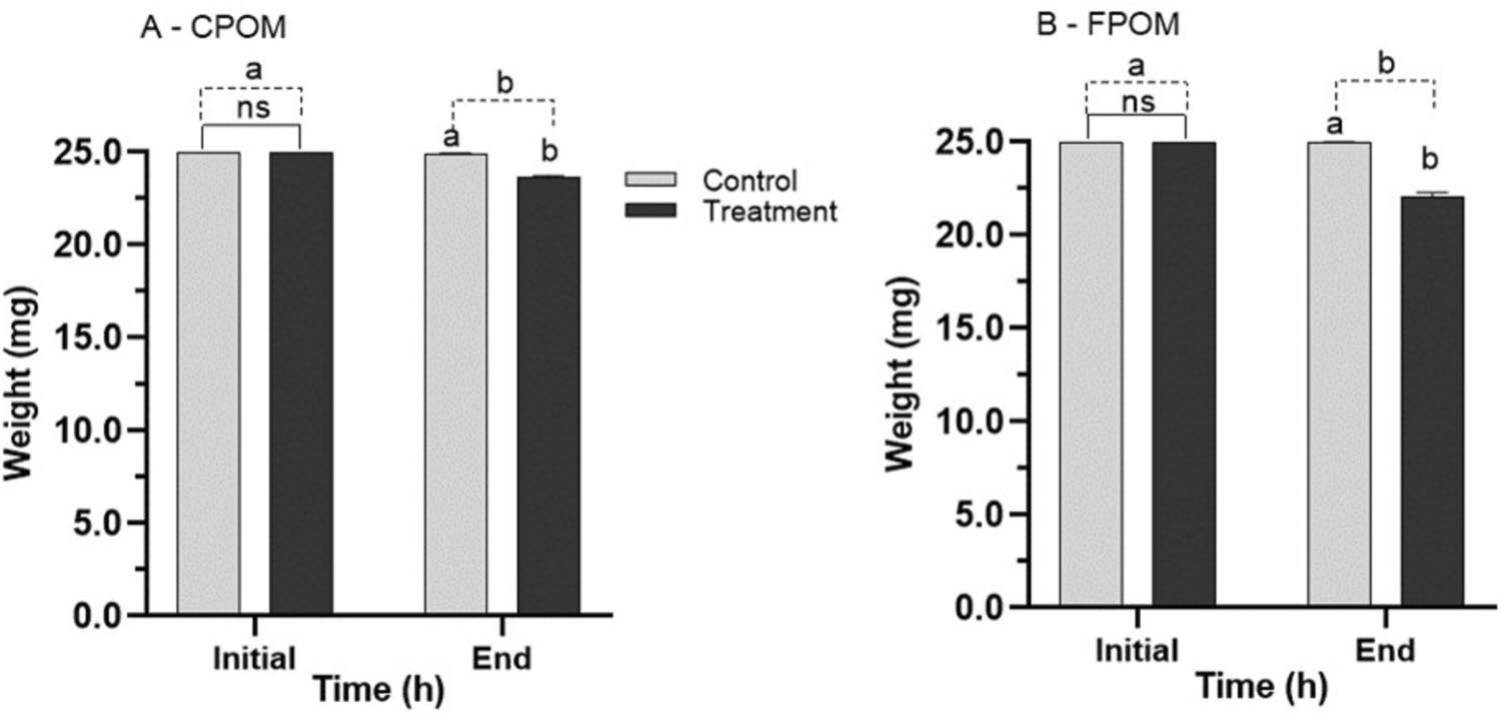
Mean ± SD of weight (mg) of coarse particulate organic matter (CPOM) (**A**) and fine particulate organic matter (FPOM) (**B**) after 48-h exposure to nymphs of *Americabaetis alphus*. Means with different letters within each time—statistical difference between treatment (with nymphs) and control (no nymphs) (*p *< 0.05); Different letters for each time (dotted stroke) – statistical difference between the times (*p* < 0.05); NS = no significant difference (*p* > 0.05)

**Fig. 7:**
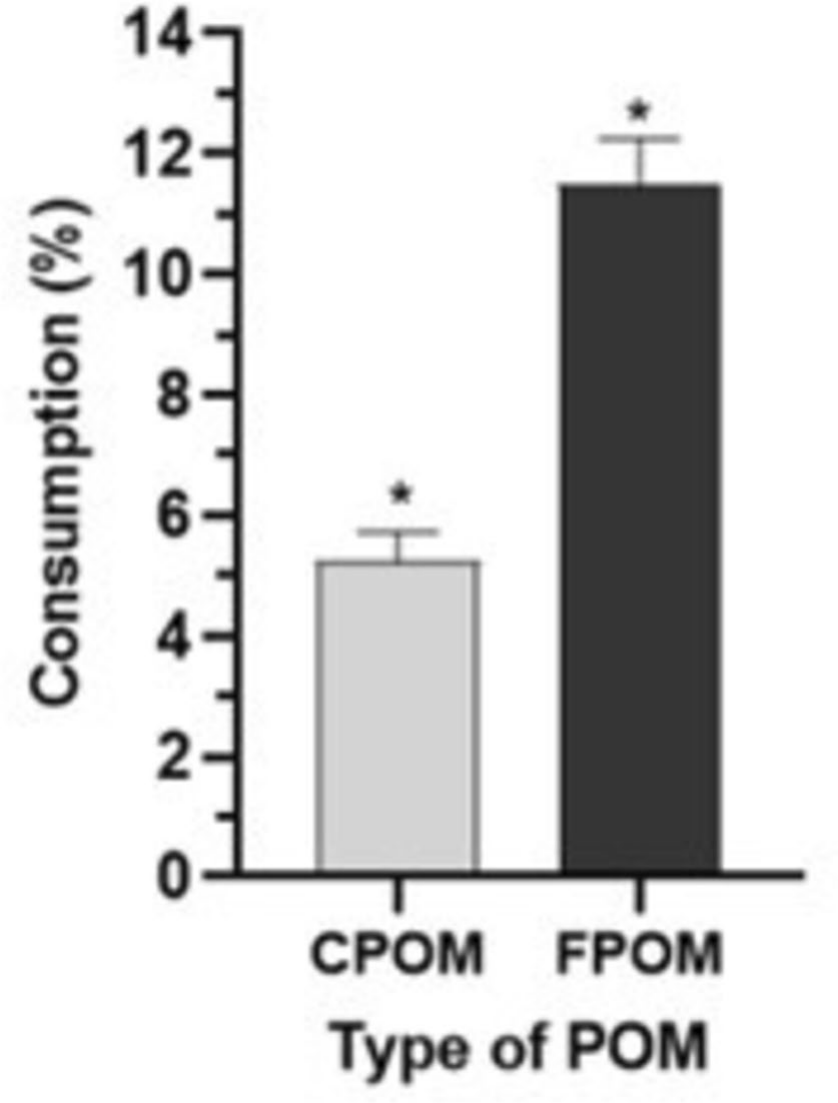
Percentage (%) consumption of fine particulate organic matter (FPOM) and coarse particulate organic matter (POM) by nymphs of *Americabaetis alphus* in 48 h. * = significant difference (*p* < 0.05)

The weights of FPOM and CPOM were equal in the control and the treatment at T_0_ T0 (*p* = 0.99, p = 0; *p* = 0.99, q = 0) (Figs. 6A, 6B). The decrease in the weight of FPOM from T_0_ to 48 h in treatment was significantly greater (*p* = 0.0001, *q *= 21.5) than the CPOM weight decrease (*p *= 0.0001, *q* = 20.02) (Fig. 6A, B). The final mean weight of CPOM was 23.6 ± 0.25 mg, whereas it was 22.1 ± 0.37 mg for FPOM (Fig. 6A, B). Consumption of FPOM (11.5 ± 1.51%) was significantly higher (*p *= 0.0001, t = 6.85) than consumption of CPOM (5.2 ± 1.02%) (Fig. 7).

## Discussion

The nymphs of *A. alphus* showed a preference for feeding on SSS rather than AS, as well as a preference for FPOM (smaller size) over CPOM (larger size) of *S. humboldtiana.* These results reject the hypothesis that *A. alphus* nymphs have a feeding preference under AS, but confirm the hypothesis that they prefer FPOM.

Studies involving experimental feeding approaches with Ephemeroptera associated with substrates of different types of material types are still scarce. Many of the previous records of Ephemeroptera feeding preference are based on "water-based cultures", e.g., feeding preference between diatoms and detritus by *Ephemerella ignita* (Ephemerellidae) (Rosillon 1988), preference between leaves, fecal material from other insects, and debris by *Drunella grandis* and *Ephemerella inermis* (both Ephemerellidae) (Shepard and Minshall 1984), preference between green algae and leaves of *Typha* sp. by *Cloeon dipterum* (Baetidae) (Šupina et al. 2016), and FPOM and CPOM preference by *C. dipterum* (Banegas et al. 2020).

The preference found for the SSS can be attributed to several factors. Stainless steel is a good conductor of heat, which promotes more stable conditions for periphyton development, particularly bacterial colonization (Palmer et al. 2007). In our study, the SSS exhibited higher bacterial biomass and chlorophyll-a concentration than AS. Metal substrates, such as stainless steel, enhance microbial adhesion, resulting in a denser and more diverse periphyton (Flemming and Wingender 2010). This greater adhesion occurs due to substrate surface roughness (Agostini et al*.*^2018^), which facilitates the adsorption and absorption of organic and inorganic particles (Patil and Anil 2005).

Another factor that may influence adhesion to the stainless steel is the presence of zinc. Zinc is an essential element in the metabolism of bacteria, acting in the growth and enzymatic activity of heterotrophic bacteria (Bong et al. 2010). The role of this element favors the development of bacterial biofilm (Agostini et al. 2019), thereby promoting greater bacterial adhesion and the formation of richer and more complex biofilms. Thus, this results in greater availability of food resources for the nymphs of *A. alphus*. In addition, bacteria are the first organisms to establish themselves during biofouling, influencing the adhesion of other microorganisms (e.g., algae, fungi, and protozoa) (Agostini et al*.*^2018^). This explains the high bacterial biomass on SSS, resulting in a higher concentration of chlorophyll-a than on AS, and making SSS a food source with a denser and more nutritious biofilm. Despite the high diversity of periphyton on plastic surfaces (Vannini et al. 2021), its overall density can be relatively low when compared to other surfaces.

The preference for SSS is also explained by the high frequency of diatoms on this material and the exclusive of green algae onAS. Diatoms rapidly colonize surfaces in freshwater, forming a dense periphyton community that supports herbivore populations, while green algae do not have the same degree of abundance in benthic substrates (Bothwell et al. 1996; Felisberto and Rodrigues 2012). Additionally, diatoms are known to be more nutritious than green algae, as they have high levels of polyunsaturated fatty acids, which are essential for the development and growth of many aquatic organisms (Brett and Müller‐Navarra 1997). Their cell walls are composed of silica, which, despite being rigid, are more easily degraded than the cellulose-rich cell walls of green algae (Cattaneo and Mousseau 1995). Thus, the presence of diatoms provides higher nutritional value and greater ease of digestion, making the SSS more attractive to *A. alphus* nymphs*.* Finally, in a field study, Hamilton and Hugh (1983) reported that, apart from debris, diatoms are the only resource with relative importance in the diet of species of Baetidae.

Although artificial substrates do not fully reproduce the structural complexity and physicochemical properties of natural benthic substrates, they are widely used as standardized tools to assess periphyton development and the responses of aquatic consumers (Cattaneo and Amireault 1992; Larned 2010). In addition, artificial substrates, including plastics, can support high biological diversity when compared with other environmental compartments (e.g., rocks, water, and sediment) (Vanini et al. 2021). In natural freshwater environments, despite the heterogeneity of available substrates, diatoms are consistently among the dominant components of periphyton, especially in lotic systems, due to their rapid colonization and high attachment capacity (Bothwell et al. 1996; Felisberto & Rodrigues 2012). Thus, the preference of *A. alphus* nymphs for substrates supporting diatom-dominated periphyton, observed under laboratory conditions, may reflect feeding patterns that also occur in natural habitats, supporting the use of artificial substrates as functional analogues of natural hard substrates.

The finding of *A. alphus* preferring ingests FPOM was expected and corroborates the results obtained by Banegas et al. (2020) for *C. dipterum*. In an experimental approach, the authors found a higher rate of consumption of FPOM consumption than of CPOM. Another approach used by Banegas et al. (2020) aimed to analyze the stomach contents of nymphs of *C. dipterum* collected in the field. When examining stomach contents and identifying eight categories of food resources, FPOM represented the highest frequency of occurrence. Other frequently occurring resources in the stomach contents of *C. dipterum* nymphs were green algae and diatoms. When comparing these results with those of our study, both species prefer the smaller FPOM. However, *A. alphus* prefers a substrate with the presence of diatoms, while in *C. dipterum* there is a higher frequency of green algae in the stomach contents.

The dietary preference for FPOM is well recognized in the literature. Smaller particles are easier to ingest and process (Cresswell and Van Hengstum 2021) and require less capture effort for *A. alphus*. In addition, FPOM is commonly composed of a higher proportion of fatty acids and proteins, making it more nutritious and attractive to organisms (Wotton 2020). This combination of ease of ingestion and higher nutritional value makes nymphs generally prefer FPOM, although they also feed on CPOM (Banegas et al. 2020) using specialized mouthparts (Jacobus et al. 2019).

The results of our study demonstrated that *A. alphus* nymphs exhibit a clear feeding preference for periphyton adhering to SSS and for a finer size of particulate organic matter from *S*. *humboldtiana*. However, the study evaluated the feeding preferences in two distinct experiments conducted separately, to verify the potential of these resources in the diet of the nymphs. In this sense, the outcomes of this study raise new questions to be addressed. Studies with this type of approach are important to clarify the functional role of species of Ephemeroptera in aquatic ecosystems and to understand their distribution that is mainly related to their preference for substrate type and sensitivity to environmental factors (Arimoro and Muller 2010; Mebarki et al. 2017). Thus, we recommend evaluating feeding preferences integrated experimental designs that combine different substrates and distinct classes of particulate organic matter, in order to better understand the primary and secondary roles of functional feeding groups. We also highlight the need to experimentally evaluate other Baetidae species, given the scarcity of information, which is largely concentrated Ephemerellidae species.

## Data Availability

The data that support the findings of this study are available from the corresponding author upon reasonable request.

## References

[CR1] Agostini VO, Macedo AJ, Muxagata E, Pinho GLL (2019) Surface coatings select their micro and macrofouling communities differently on steel. Environ Pollut 254:113086. 10.1016/j.envpol.2019.113086 31479812 10.1016/j.envpol.2019.113086

[CR2] Agostini VO, Muxagata E, Pinho GLL et al (2021) Bacteria-invertebrate interactions as an asset in developing new antifouling coatings for man-made aquatic surfaces. Environ Pollut 271:116284. 10.1016/j.envpol.2020.116284 33360655 10.1016/j.envpol.2020.116284

[CR3] Arimoro FO, Muller WJ (2010) Mayfly (Insecta: Ephemeroptera) community structure as an indicator of the ecological status of a stream in the Niger Delta area of Nigeria. Environ Monit Assess 166:581–594. 10.1007/s10661-009-1025-3 19543701 10.1007/s10661-009-1025-3

[CR4] Banegas BP, Casset MA, Silvera A, Rocha L (2020) Mouthpart morphology and food habits of a Pampean population of *Cloeon dipterum* (Linnaeus, 1761) (Ephemeroptera: Baetidae). Ann Limnol - Int J Lim 56:21. 10.1051/limn/2020019

[CR5] Bao S, Jin L, Wu Q et al (2023) Effects of interspecific interactions on aquatic macrophyte litter decomposition and its influencing factors. ACS EST Water 3:3755–3766. 10.1021/acsestwater.3c00256

[CR6] Bong CH, Malfatti F, Azam F, Suzuki S (2010) The effect of zinc exposure on the bacteria abundance and proteolytic activity in seawater. Interd. Studies Environ Chem — Biological Responses to Contaminants. Terrapub, Tokyo, pp 57–63

[CR7] Bothwell ML, Lowe RL, Stevenson RJ (1996) Algal ecology: freshwater benthic ecosystems. Academic Press, San Diego

[CR8] Boyero L, Pearson RG, Albariño RJ et al (2020) Identifying Stream Invertebrates as Plant Litter Consumers. In: Bärlocher F, Gessner MO, Graça MAS (eds) Methods to Study Litter Decomposition. Springer International Publishing, Cham, pp 455–464. 10.1007/978-3-030-30515-4_50

[CR9] Brett M, Müller-Navarra D (1997) The role of highly unsaturated fatty acids in aquatic foodweb processes. Freshw Biol 38:483–499. 10.1046/j.1365-2427.1997.00220.x

[CR10] Cattaneo A, Amireault MC (1992) How artificial are artificial substrata for periphyton? J North Am Benthol Soc. 10.2307/1467389

[CR11] Cattaneo A, Mousseau B (1995) Empirical analysis of the removal rate of periphyton by grazers. Oecologia 103:249–254. 10.1007/BF00329087 28306780 10.1007/BF00329087

[CR12] Chuang Y-L, Yu S-F, Lin H-J (2014) Dietary variation and food selection by mayfly grazers in a subtropical mountain stream. Zool Stud 53:54. 10.1186/s40555-014-0054-y

[CR13] Cresswell JN, Van Hengstum PJ (2021) Habitat partitioning in the marine sector of karst subterranean estuaries and Bermuda’s marine caves: benthic foraminiferal evidence. Front Environ Sci 8:594554. 10.3389/fenvs.2020.594554

[CR14] Da-Silva ER, Salles FF (2024) Insetos do Brasil: Diversidade e Taxonomia. In: Rafael JA, Melo GAR, Carvalho CJBde, Casari S, Constantino R (eds) 2nd edn. Instituto Nacional de Pesquisas da Amazônia, Manaus. 10.61818/56330464

[CR15] Domínguez E, Molineri C, Pescador ML et al (2006) Aquatic Biodiversity in Latin America: Ephemeroptera of South America. In: Adis J, Arias JR, Rueda-Delgado G, Wantzen KM (eds). Sofia–Moscow. https://books.pensoft.net/books/8235

[CR16] Domínguez E, Emmerich D, Molineri C, Nieto C (2023) Ephemeroptera. In: Claps LE, Roig-Juñent SA, Morrone JJ (eds) Biodiversidad de Artrópodos Argentinos, 1st edn. Editorial INSUE – UNT, San Miguel de Tucumán – Argentina, p 531

[CR17] Edmunds JGE, Jensen SL, Berner L (1976) The mayflies of North and Central America. University of Minnesota Press, Minnesota

[CR18] Eisler R (1998) Copper hazards to fish, wildlife and invertebrates: a synoptic review. U.S. Department of the Interior, Geological Survey, Laurel, MD

[CR19] Felisberto SA, Rodrigues L (2012) Dinâmica sucessional de comunidade de algas perifíticas em um ecossistema lótico subtropical. Rodriguésia 63:463–473. 10.1590/S2175-78602012000200018

[CR20] Flemming H-C, Wingender J (2010) The biofilm matrix. Nat Rev Microbiol 8:623–633. 10.1038/nrmicro2415 20676145 10.1038/nrmicro2415

[CR21] França RCS, Lopes MRM, Ferragut C (2011) Structural and successional variability of periphytic algal community in a Amazonian lake during the dry and rainy season (Rio Branco, Acre). Acta Amaz 41:257–266. 10.1590/S0044-59672011000200010

[CR22] Gonçalves FFJ, Martins RT, Ottoni BMP, Couceiro CRM (2014) Uma visão sobre a decomposição foliar em sistemas aquáticos brasileiros. In: Hamada N, Nessimian JK, Querino RB (eds) Insetos aquáticos: Biologia, Ecologia e Taxonomia. . Editora do INPA, Manaus, pp 89–116

[CR23] Graça MAS, Bärlocher F, Gessner MO (eds) (2005) Methods to Study Litter Decomposition. Springer-Verlag, Berlin/Heidelberg

[CR24] Gutiérrez Y, Llano CA (2015) First record of *Americabaetis alphus* (Insecta: Ephemeroptera: Baetidae)from Colombia. Rev Colomb Entomol 41:147–148

[CR25] Hamilton HR, Hugh FC (1983) The seasonal food habits of mayfly (Ephemeroptera) nymphs from three Alberta, Canada, streams, with special reference to absolute volume and size of particles ingested. Arch Hydrobiol 65(2/3):197–234

[CR26] Jacobus LM, Macadam CR, Sartori M (2019) Mayflies (Ephemeroptera) and their contributions to ecosystem services. Insects 10:170. 10.3390/insects10060170 31207933 10.3390/insects10060170 PMC6628430

[CR27] Kaltenbach T, Gattolliat J-L (2019) The tremendous diversity of *Labiobaetis* Novikova & Kluge in Indonesia (Ephemeroptera, Baetidae). ZK 895:1–117. 10.3897/zookeys.895.38576 10.3897/zookeys.895.38576 PMC6906171 31844411

[CR28] Larned ST (2010) A prospectus for priphyton: recent and future ecological research. J N Am Benthol Soc 29:1. 10.1899/08-063.1

[CR29] Liu J, Dazzo FB, Glagoleva O et al (2001) CMEIAS: a computer-aided system for the image analysis of bacterial morphotypes in microbial communities. Microb Ecol 41:173–194 11391457 10.1007/s002480000004

[CR30] Lopretto EC, Tell G (1995) Ecosistemas de Aguas Continentales: Metodologias para su Estudio. Ediciones SUR, La Plata - Argentina

[CR31] Mackinney G (1941) Absorption of light by Chlorophyll solutions. J Biol Chem 140:315–322. 10.1016/S0021-9258(18)51320-X

[CR32] Maltchik L, Stenert C, Spies MR, Siegloch AE (2009) Diversity and distribution of Ephemeroptera and Trichoptera in Southern Brazil Wetlands. J Kans Entomol Soc 82:160–173. 10.2317/JKES808.04.1

[CR33] Marks JC (2019) Revisiting the fates of dead leaves that fall into streams. Annu Rev Ecol Evol Syst 50:547–568. 10.1146/annurev-ecolsys-110218-024755

[CR34] Martín-Rodríguez AJ, Babarro JMF, Lahoz F et al (2015) From broad-spectrum biocides to quorum sensing disruptors and mussel repellents: antifouling profile of alkyl Triphenylphosphonium salts. PLoS ONE 10:e0123652. 10.1371/journal.pone.0123652 25897858 10.1371/journal.pone.0123652 PMC4405350

[CR35] McShaffrey D, McCafferty WP (1991) Ecological association of the mayfly *Ephemerella needhami* (Ephemeroptera: Ephemerellidae) and the green alga *Cladophora* (Chlorophyta: Cladophoraceae). J Freshwater Ecol 6:383–394. 10.1080/02705060.1991.9665318

[CR36] Mebarki M, Taleb A, Arab A (2017) Environmental factors influencing the composition and distribution of mayfly larvae in northern Algerian wadis (regional scale). Revec 72:303–313. 10.3406/revec.2017.1893

[CR37] Merritt RW, Cummins K (1996) An introduction to the aquatic insects of North America. Kendall/Hunt Publishing Company, Dubuque

[CR38] Morales MLP, Figurelli GP, Oleinski B et al (2024) Antifouling activity of aquatic macrophyte extracts on estuarine bacterial biofilms. Chem Ecol 40:388–406. 10.1080/02757540.2024.2321990

[CR39] Agostini V O, José Macedo A, Muxagata E (2018) O papel do biofilme bacteriano no acoplamento bentopelágico, durante o processo de bioincrustação. RL 19:23–41. 10.31514/rliberato.2018v19n31.p23

[CR40] Palmer J, Flint S, Brooks J (2007) Bacterial cell attachment, the beginning of a biofilm. J Ind Microbiol Biotechnol 34:577–588. 10.1007/s10295-007-0234-4 17619090 10.1007/s10295-007-0234-4

[CR41] Paranhos R (1996) Alguns métodos para ánalise de água. Cadernos Didáticos, Rio de Janeiro

[CR42] Patil JS, Anil AC (2005) Biofilm diatom community structure: influence of temporal and substratum variability. Biofouling 21:189–206. 10.1080/08927010500256757 16371339 10.1080/08927010500256757

[CR43] Rosillon D (1988) Food preference and relative influence of temperature and food quality on life history characteristics of a grazing mayfly, *Ephemerella ignita* (Poda). Can J Zool 66:1474–1481. 10.1139/z88-214

[CR44] Salinas-Jimenez LG, Dias LG, Román-Valencia C (2025) A new species of *Americabaetis* (Baetidae: Ephemeroptera) from Andean region, Colombia. Pap Avulsos Zool 65:e202565012. 10.11606/1807-0205/2025.65.012

[CR45] Salles FF, Francischetti CN, Roque FDO et al (2003) Levantamento preliminar dos gêneros e espécies de baetidae (Insecta: Ephemeroptera) do estado de São Paulo, com ênfase em coletas realizadas em córregos florestados de baixa ordem. Biota Neotrop 3:1–7. 10.1590/S1676-06032003000200011

[CR46] Salles FF, Raimundi EA, Boldrini R, Souza-Franco GM (2010) The genus Americabaetis Kluge (Ephemeroptera: Baetidae) in Brazil: new species, stage description, and key to nymphs. Zootaxa 2560:. 10.11646/zootaxa.2560.1.2

[CR47] Sartori M, Brittain JE (2015) Order Ephemeroptera. In: Thorp and Covich’s freshwater invertebrates, 4th edn. Elsevier, pp 873–891. 10.1016/B978-0-12-385026-3.00034-6

[CR48] Shepard RB, Minshall GW (1984) Selection of fine-particulate foods by some stream insects under laboratory conditions. Am Midl Nat 111:23. 10.2307/2425538

[CR49] Shimano Y, Salles FF, Faria LRR et al (2012) Distribuição espacial das guildas tróficas e estruturação da comunidade de Ephemeroptera (Insecta) em córregos do Cerrado de Mato Grosso, Brasil. Iheringia, Sér Zool 102:187–196. 10.1590/S0073-47212012000200011

[CR50] Smeda-Pienaar K, Kaambo E, Africa CWJ (2017) Bacterial morphotype grading for periodontal disease assessment. BDJ Open 3:16011. 10.1038/bdjopen.2016.11 29607072 10.1038/bdjopen.2016.11 PMC5842865

[CR51] Šupina J, Bojková J, Boukal DS (2016) Influence of food availability, predation risk and initial body size on growth and maturation of *Cloeon dipterum* (Ephemeroptera: Baetidae). Zoosymposia 11:53–64. 10.11646/zoosymposia.11.1.9

[CR52] Telöken F, Albertoni EF, Palma-Silva C (2011) Leaf degradation of *Salix humboldtiana* Willd. (Salicaceae) and invertebrate colonization in a subtropical lake (Brazil). Acta 23:30–41. 10.4322/actalb.2011.016

[CR53] Tonin AM, Ubiratan Hepp L, Gonçalves JF (2018) Spatial variability of plant litter decomposition in stream networks: from litter bags to watersheds. Ecosystems 21:567–581. 10.1007/s10021-017-0169-1

[CR54] Vannini C, Rossi A, Vallerini F et al (2021) Microbial communities of polyhydroxyalkanoate (PHA)-based biodegradable composites plastisphere and of surrounding environmental matrix: a comparison between marine (seabed) and coastal sediments (dune sand) over a long-time scale. Sci Total Environ 764:142814. 10.1016/j.scitotenv.2020.142814 33129544 10.1016/j.scitotenv.2020.142814

[CR55] Wotton RS (2020) The biology of particles in aquatic systems, 2nd edn. CRC Press

